# Long-term Follow up of Mesenchymal Hamartoma of Liver- Single Center Study

**DOI:** 10.4103/1319-3767.74449

**Published:** 2011

**Authors:** Anand Pandey, Ajay N. Gangopadhyay, Shiv P. Sharma, Vijayendra Kumar, Dinesh K. Gupta, Saroj C. Gopal, Shashikant C. Patne

**Affiliations:** Department of Pediatric Surgery, Institute of Medical Sciences, Banaras Hindu University, Varanasi - 221 005, U.P., India; 1 Department of Pathology, Institute of Medical Sciences, Banaras Hindu University, Varanasi - 221 005, U.P., India

**Keywords:** Hamartoma, liver tumor, mesenchymal hamartoma of liver, pediatric liver tumors

## Abstract

**Background/Aim:**

Mesenchymal hamartoma of liver (MHL) is a rare liver tumor of childhood. About 200 cases have been reported till now. Most of the work on MHL is limited to case reports and there are not many long term follow-up studies. We present our 20 years of experience with this uncommon entity. This study aims to highlight clinical features, diagnosis and treatment of MHL.

**Materials and Methods:**

All patients with a diagnosis of MHL in last 20 years were included in this retrospective study. The patients were evaluated clinically, radiologically and pathologically.

**Results:**

The total number of patients with a diagnosis of MHL was nine. Mean age of the patients was 19.89 ± 2.75 months. Right lobe was involved in eight patients. The prominent clinical features were distension of abdomen and anorexia. Surgical options used were hepatic lobectomy, wedge resection and enucleation. Histopathology of the specimens showed cysts of variable size with normal hepatocytes, bile ducts and connective tissue stroma. Overall mortality was one (11.11%).

**Conclusion:**

MHL is a benign tumor that can present with various clinical features. It should be differentiated carefully from other liver masses especially malignant ones. The diagnosis can be made with the help of radiology and histopathology. Adequate resection is curative in most of the cases and long-term follow up is satisfactory.

Mesenchymal hamartoma of liver (MHL) is a rare liver tumor of childhood.[1] It is the second most common benign liver tumor in children after hemangioma.[2] About 200 cases have been reported till now.[3] Primary hepatic neoplasms in children account for between 0.5 and 2.0% of all pediatric neoplasms. They are a diverse group of epithelial and mesenchymal tumors. The incidence can vary with patient age. About two thirds of primary hepatic neoplasms are malignant,[4] rest being benign. Most of the work on MHL is limited to case reports and long-term follow up studies are not much. We present our 20 years of experience with this uncommon entity. This study aims to highlight clinical features, diagnosis and treatment of MHL.

## MATERIALS AND METHODS

This was a retrospective study carried out in the department from June 1987 to June 2007. All patients with a diagnosis of MHL were included in the study. The complaints of abdominal pain and distension were evaluated by ultrasonography (USG) of the abdomen. Complete blood counts, renal and liver function test were done preoperatively to assess the fitness of the patient. Alfa feto protein (AFP) was done for follow-up purpose.

The patients were managed by operation, which consisted of hepatic lobectomy, segmental hepatic resection or enucleation. The patients were followed up for the response to the treatment and recurrence.

## RESULTS

The total duration of the study period was 20 years. The total number of patients with a diagnosis of MHL was nine. Mean age of the patients was 18.89 ± 14.75 months (ranges from one month to four years). The male to female ratio was 8:1. MHL involved the right lobe in eight (88.88%) patients, whereas only one (11.11%) patient had involvement of left lobe. The clinical features noted were distension of abdomen in all patients (100%), anorexia in eight (88.88%), pain in abdomen in six (66.67%) and respiratory distress in four (44.44%) patients. USG of the abdomen was able to detect the liver mass in all patients. It showed multiple cysts in seven (77.78%) patients, and the rest two (22.22%) had predominantly solid appearing lump. Serum AFP was within normal limits in all the patients except one. Renal and liver functions were normal in all the patients.

**Figure 1:**
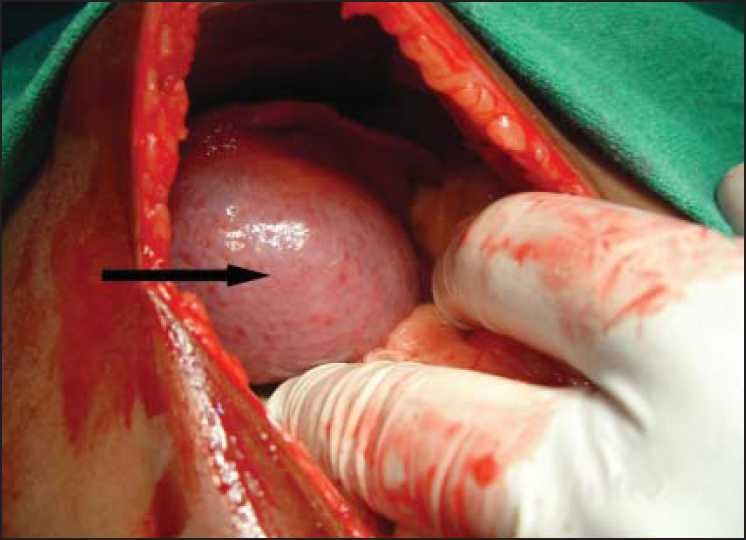
Intraoperative photograph showing mesenchymal hamartoma in the right lobe of liver (marked by arrow). The hemartoma is differentiated from the normal liver color by its dull greyish color

All patients were managed by surgery [Figure 1] which included right or left hepatic lobectomy in three (33.33%) patients, wedge resection in three (33.33%) patients, enucleation in two (22.22%) patients and partial hepatic lobectomy in one (11.11%) patients. One patient had a cardiac arrest during procedure, due to severe hypovolemia. All the remaining patients faired well in the postoperative period. The mean size of the liver mass was 179.22 ± 139.16 cm^2^ (40-418 cm^2^). The complication of the operation included biliary leak in three (33.33%) patients which stopped spontaneously in seven to ten days. No other specific complication was noted.

**Figure 2:**
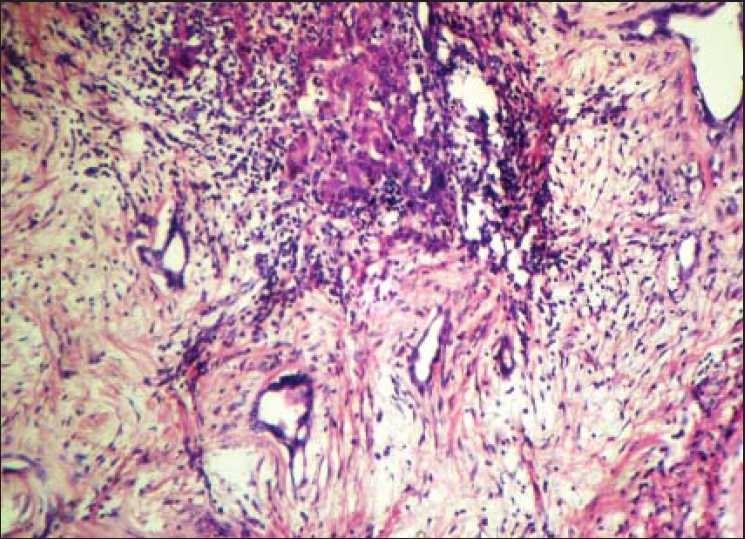
Dense fibrous tissue along with collection of inflammatory cells and cords of hepatocytes (H and E, ×100)

**Figure 3:**
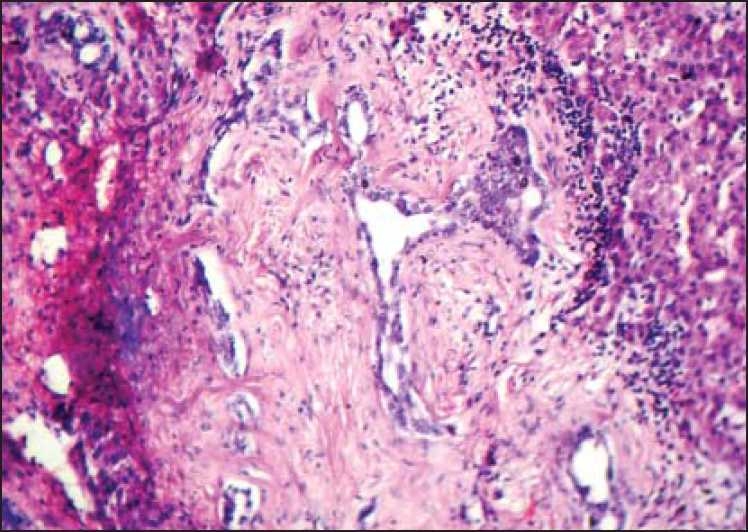
Variably dilated biliary ductular channels surrounded by the dense fibrous tissue, collection of inflammatory cells and cords of hepatocytes (H and E, ×100)

Histopathology of the specimens showed cysts of variable size with normal hepatocytes, bile ducts and connective tissue stroma [Figures 2 and 3]. There was no evidence of malignancy in any specimen.

Overall mortality was one (11.11%). All patients had satisfactory weight and height gain according to the CDC growth charts (CDC growth charts United States: http/www.cdc.gov) in the follow up. The mean duration of follow up was 6.00 ± 2.78 years (range 1 to 9 years). There was no complication noted in the follow-up period. There has been no recurrence up till now in any of the patients.

## DISCUSSION

MHL accounts for 22% of all benign liver tumors of childhood and they generally present in patients less than 2 years of age.[5] It has been described in the literature by various names like pseudocystic mesenchymal tumor, giant cell lymphangioma, cystic hamartoma, bile cell fibroadenoma, hamartoma, and cavernous lymphangiomatoid tumor.[6] The first definitive description of MHL was by Edmondson.[7]

Various theories for its occurrence have been suggested such as ductal plate malformation or toxic injury to the fat-storing (Ito) cells of the immature liver,[8] and localized vascular insult to the developing fetal liver.[9] It has been found to be associated with abnormalities of chromosome 19.[10]

The diagnosis of MHL may be incidental but typically it presents with abdominal distension with or without an abdominal mass. Abdominal pain, anorexia, vomiting, and poor weight gain have also been reported.[11,12] These clinical features were also noted in our series and same features were noticed by Karpelowsky *et al*[13] in their series of 17 patients. The examination of the abdomen reveals a large, nontender, firm, and smooth liver mass. Visible engorged veins over the anterior abdominal wall and lower limb edema due to inferior vena cava compression may sometimes be noticed.[6,12] Respiratory distress noticed in our patients may have been caused by the upward pressure on the diaphragms.[6]

Liver function tests including AFP are usually normal, but may be mildly deranged. Some patients may have mild elevation in serum AFP concentration which was seen in one patient in our series, but it decreases to normal after complete tumor resection.[12] USG of the abdomen, computerized tomography (CT) scan and magnetic resonance imaging (MRI) have been used to confirm the diagnosis. USG finding of round hyperechoic parietal nodules within the cystic spaces of the hamartomas has been found to be specific for MHL.[14] On an unenhanced CT, MHL usually has a heterogeneous appearance. The stromal elements appear hypoattenuating, whereas the cystic component has water attenuation. After intravenous administration of contrast material, the stromal component enhances. The MRI appearance of mesenchymal hamartoma varies depending on the presence of stromal elements and the protein content of the fluid.[15] CT scan was not done for any patient. Although USG could be diagnostic, CT scan remains essential in planning any liver resection due to better anatomic delineation. In our setup, because of cost constraints, we go for CT scan only if USG is inconclusive about resectability of the tumor. The findings of fine needle aspiration may be nonspecific but it may rule out many other diagnostic possibilities and increases the preoperative capacity of clinical and image studies, leading to a more rational therapeutic decision.[16]

Traditionally, the surgical treatment has been complete tumor excision, either nonanatomically with a rim of normal tissue or as an anatomic hepatic lobectomy. If the tumor is considered unresectable, the surgical options include enucleation and marsupialization.[6] Lobectomy was the preferred option both in our and Karpelowsky series.[13] Very rarely liver transplantation may be needed.[12] Despite the opinion of conservative management for selected cases,[11] we favor surgery, as there is a possibility of development of malignancy in MHL.[12] Recently, laparoscopic liver resection for MHL has been reported with successful result.[17]

About 75% of MHL occur in the right lobe of the liver; the rest are found in the left lobe or involve both lobes.[6,12] In our series, the right lobe was involved in more than 85% of patients whereas Karpelowsky had 70% involvement of the right lobe.

Pathologically, MHL is characterized by the presence of connective or mesenchymal tissue stroma, bile ducts, serous cysts, hepatocytes and angiomatous components.[18] Outcome of the treatment is usually satisfactory.

To conclude, MHL is a benign tumor that can present with various clinical features. It should be differentiated carefully from other liver masses especially malignant ones. The diagnosis can be made with the help of radiology and histopathology. Adequate resection is curable in most of the cases and long-term follow up is satisfactory.
